# Thermodynamic Solution Properties of a Biodegradable Chelant (L-glutamic-N,N-diacetic Acid, L-GLDA) and Its Sequestering Ability toward Cd^2+^

**DOI:** 10.3390/molecules26237087

**Published:** 2021-11-23

**Authors:** Clemente Bretti, Roberto Di Pietro, Paola Cardiano, Olivia Gomez-Laserna, Anna Irto, Gabriele Lando, Concetta De Stefano

**Affiliations:** 1 Department of Chemical, Biological, Pharmaceutical and Environmental Sciences, University of Messina, Viale Ferdinando Stagno d’Alcontres 31, I-98166 Messina, Italy; cbretti@unime.it (C.B.); robdipietro@unime.it (R.D.P.); pcardiano@unime.it (P.C.); airto@unime.it (A.I.); cdestefano@unime.it (C.D.S.); 2 Department of Analytical Chemistry, University of the Basque Country (EHU/UPV), Barrio Sarriena s/n Leioa, E-48080 Bilbao, Spain; olivia.gomez@ehu.eus

**Keywords:** cadmium, L-glutamic-N,N-diacetic acid, sequestering ability, potentiometry, thermodynamics, pL_0.5_, sustainable molecules

## Abstract

The thermodynamics of the interaction of L-glutamic-N,N-diacetic acid (GLDA) with protons was studied potentiometrically at different temperatures, ionic strengths and ionic media. Four protonation constants and corresponding enthalpy changes occurred at infinite dilution together with temperature and ionic strength coefficients. The medium effect was also interpreted in terms of the formation of weak complexes between the ligand and the cations of supporting electrolytes, resulting in a greater tendency of GLDA to chemically interact with Na^+^ rather than K^+^ and, in turn, (CH_3_)_4_N^+^. Formation constants of GLDA with Cd^2+^ were determined in NaCl_(aq)_ at different ionic strength values. Five complex species were found, namely CdL^2−^, CdHL^−^, CdH_2_L^0^_(aq)_, Cd_2_L^0^_(aq)_, and Cd(OH)L^3−^, whose formation constant values at infinite dilution were log *β* = 12.68, 17.61, 20.76, 17.52, and 1.77, respectively. All the species results were relevant in the pH range of natural waters, although the Cd_2_L^0^_(aq)_ was observed only for *C*_Cd_ ≥ *C*_GLDA_ and concentrations of >0.1 mmol dm^−3^. The sequestering ability of GLDA toward Cd^2+^, evaluated by means of pL_0.5_, was maximum at pH~10, whereas the presence of a chloride containing a supporting electrolyte exerted a negative effect. Among new generation biodegradable ligands, GLDA was the most efficient in Cd^2+^ sequestration.

## 1. Introduction

**Scheme 1:**
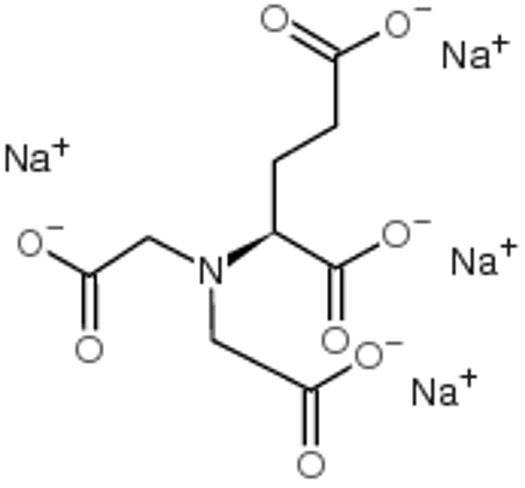
Structure of L-glutamic-N,N-diacetic acid (L-GLDA) tetrasodium salt.

The demand for biodegradable products coming from sustainable and renewable sources, for research and industrial applications, has grown considerably, with the aim to alleviate the issues related to persistent pollutants in the environment. Among chemicals widely employed in many fields of industry, chelating agents are used to efficiently control metal ions in several contexts such as household cosmetics and detergents, pharmaceuticals, the pulp and paper industry, textiles, and so forth [1,2,3]. Chelating agents, due to their ability to form stable complexes with metal cations, as well as their industrial applications, are responsible for various negative effects on the environment since they can mobilize radioactive metal ions or other contaminating metal cations, increasing their bioavailability, contributing to the eutrophication of water by dissolving insoluble phosphates. Most significantly, they may increase the bioavailability of dangerous heavy metals, since sometimes metal–ligand complexes such as Cu(II)-EDTA and Cd(II)-EDTA are much more toxic than free metal cations. Furthermore, the low biodegradability of the most employed chelating agents, as well as their tendency to accumulate as such in the environment, has become an important issue. L-glutamic-N,N-diacetic acid (L-GLDA, Scheme 1) belongs to the new generation of environmentally friendly and non-toxic chelating agents [4,5,6,7]. Whereas traditional chelating agents like EDTA (ethylenediaminetetraacetic acid) and DTPA (diethylenetriaminepentaacetic acid) are negligibly biodegradable, L-GLDA was found to be readily biodegradable, according to the OECD test guidelines for chemicals [8,9]. Low toxicity (LD_50_ > 2000 mg kg^−1^) makes GLDA safe for humans. GLDA can also be considered bio-based since its synthesis is based on the flavor enhancer monosodium glutamate from the fermentation of corn molasses [10].

Besides the industrial applications in a variety of formulations, GLDA was successfully tested as a potential candidate for the remediation of heavy metal contaminated soils, sewage sludge and industrial sludge, to name only a few. GLDA was investigated to assess its removal efficacy and mechanisms toward Cd, Pb and Zn from real mines and polluted farmland soils [11,12]; it was evaluated as a chelating agent for Cd, Co, Cu, Zn, Ni and Cr from sewage sludge [13]; GLDA was also found to be effective in the removal of Cd, Ni and Cu from industrial sludge [14]; it was successfully employed in the washing of soil contaminated with toxic metals, in a pilot-scale experiment to simulate real remediation operation technology [15] and as a heavy metal activator to enhance phytoremediation in red soils [16]. In addition, as a mixture with citric (CIT) or ascorbic acid, GLDA was efficiently used for Cu, as well as Pb and Zn removal from rice paddy fields, respectively [17]; chemical washing with mixed chelators, GLDA and CIT, was also successfully employed to remove heavy metals (i.e., Cd, Cu, Zn, Mn, Cr) from sewage sludge [18]; a mix of biodegradable chelators was also used in soil washing for the decontamination of different kinds of soils [19,20]. As it can be inferred from the abovementioned literature, some parameters such as the pH of the solutions employed to remove contaminants may exert a significant effect on the extraction efficacy so that strongly acidic conditions should be preferred for toxic cations removal. Among toxic metal cations, as a prioritized environmental pollutant, Cd^2+^ has received great attention since it has been recognized as harmful for humans, even at low concentrations [21,22,23]. Cadmium is naturally released by volcanic emissions, but anthropogenic activities greatly increase its levels in the environment (in 2020, the global refinery production of cadmium was approximately 23,000 metric tons [24], mainly from Ni–Cd battery manufacturing, nonferrous metal refining, fossil fuel combustion, incineration of municipal waste, phosphate fertilizer production, as well as electric and electronic waste recycling [25]). Cadmium contamination is a global health concern since it is easily accumulated in plants and invertebrates, affects water and soil organisms, and displays biomagnification along some food chains, thus, indirectly harming human health [26]. Moreover, cadmium distribution among soil, plants and animals is dependent on metal availability in the specific conditions occurring in the environment so that its removal is largely related to its chemical speciation. In this light, the assessment of the speciation models of Cd^2+^ in the presence of GLDA is crucial since it can be a robust support in predicting the complex formation in specific experimental conditions. Moreover, the evaluation of species formation and relative stability, as well as the determination of the chelation thermodynamics of GLDA toward Cd^2+^, is essential to specifically design a sequestration strategy in view of practical heavy metal decontamination. Accordingly, a systematic study on the thermodynamics of the interaction of GLDA with protons, reporting data in a standard state (i.e., infinite dilution) and parameters to calculate protonation constants in different ionic media (NaCl, KCl, (CH_3_)_4_NCl, and (C_2_H_5_)_4_NI) at different temperatures and ionic strengths, is presented here. Moreover, the formation constants and sequestering ability of GLDA toward Cd^2+^ are determined in a wide range of experimental conditions and compared to data reported for other “traditional” and “new generation” chelating agents.

## 2. Materials and Methods

### 2.1. Chemicals

Tetraethylammonium iodide [(C_2_H_5_)_4_NI] was re-crystallized from methanol as described by Perrin et al. [27]. Other chemicals were purchased from Merck Italy at the highest purity available and were used without further purification. High purity L- glutamic-N,N-diacetic acid tetrasodium salt (GLDA, Dissolvine^®^ GL-PD-S), was obtained from AkzoNobel (Amsterdam) and was used without further purification. Potassium hydroxide, sodium hydroxide, tetraethylammonium hydroxide and hydrochloric acid solutions were prepared from concentrated solutions and standardized against potassium hydrogen phthalate (for bases) and sodium carbonate (for acids), previously dried in the oven at 383.15 K for 2 h. The concentration of GLDA in the solutions was determined by alkalimetric titrations. Sodium and potassium chloride solutions were prepared by weighing the solid previously dried in the oven at 383.15 for 2 h. Cadmium chloride solutions were prepared by weighing the salt and then standardized against EDTA. All solutions were freshly prepared, using grade A glassware and twice-distilled water (ρ ≥ 18 MΩ). All chemicals used are listed in Table 1.

### 2.2. Apparatus and Procedure for the Potentiometric Measurements

The acid–base properties of GLDA and its interaction with Cd^2+^ were studied by ISE-H^+^ potentiometric titrations carried out using a Metrohm 809 Titrando equipped with an automatic burette (total volume 10 cm^3^) and a combined glass electrode (Metrohm, model 6.0262.100). The estimated accuracy for both systems was ± 0.2 mV and ± 0.003 mL for e.m.f. and titrant volume readings, respectively. The apparatuses were connected to a PC and controlled by Tiamo 2.5 software to set titrant delivery, data acquisition and to check for e.m.f. stability. All titrations were carried out under magnetic stirring; presaturated N_2_ was bubbled through the solution to exclude/prevent O_2_ and CO_2_ dissolution. For each measurement 25 cm^3^ of titrand solution containing a suitable amount of GLDA (1.0 ≤ *C*_L_/mmol dm^−3^ ≤ 5.0), HCl_(aq)_ and ionic medium (NaCl_(aq)_, KCl_(aq)_ or (C_2_H_5_)_4_NI_(aq)_) was added to reach pre-established the values of pH (~2.0) and ionic strengths (0.1 ≤ *I*/mol dm^−3^ ≤ 1.0). For the measurements aiming to determine the stability constants of the Cd^2+^/GLDA system, CdCl_2_ (0.4 ≤ *c*_M_/mmol dm^−3^ ≤ 2.0, generally, *C*_M_ ≤ *C*_L_) was added into the cell. The titrand solutions were titrated with CO_2_-free standard base (NaOH_(aq)_, KOH_(aq)_ or (C_2_H_5_)_4_NOH_(aq)_) solutions up to pH~10.0 or until the electrode started drifting toward lower pH values which indicated the onset of precipitation of sparingly soluble species hardly detectable by the naked eye in the initial state. Potentiometric measurements were performed at different temperatures whose value was constantly maintained by using circulating water from a thermocryostat (model D1-G Haake). Further details on the potentiometric measurements are reported in Table 2.

### 2.3. Calculations

ESAB2M [28] was used to refine all the parameters of the acid–base potentiometric titrations (*E*^0^, *K*_w_, liquid junction potential coefficient, *j*_a_ and analytical concentration of reagents) whereas BSTAC [29] was employed for the calculation of equilibrium constants. The non-linear least square computer program LIANA was used to fit different equations [29]. ES2WC was used for the calculation of the pure model approach [30].

All equilibria, stepwise (*K*_ij_) and overall (*β*_ij_) described in this paper are expressed by the following Equations:

j M^n+^ + H_i_L^(i−4)^ = M_j_H_i_L^(i+jn−4)^ (1)

or

j M^n+^ + i H^+^ + L^4−^ = M_j_H_i_L^(i+jn−4)^ (2)

where M, H and L are the metal ion of interest, the proton and the GLDA anion (*Glda*^4−^), respectively. Distribution diagrams were obtained by using HySS [31]. Throughout the paper, uncertainties are given as a 95 % confidence interval. The conversion from the molar to the molal concentration scale was performed using the appropriate density values [32].

The ionic strength dependence of the equilibrium constants was studied in this work by means of the extended Debye–Hückel equation (EDH) and the Specific ion Interaction Theory (SIT) [33,34,35,36,37] model. Considering a generic complex formation constant, expressed in Equation (2), the equilibrium constants, as a function of the activity coefficients, may be expressed by Equation (3):

(3) $${\log\beta }_{\mathrm{ij}}{=\log\beta }_{\mathrm{ij}}^{0}{+i\;\cdot \;\log\gamma }_{H^{+}}{+j\;\cdot \;\log\;\gamma }_{M^{n+}}{+\log\gamma }_{L^{4-}}-{\;\log\gamma }_{{\mathrm{MjH}}_{i}L^{\left(i+\mathrm{jn}-4\right)}}$$

where $\beta_{\mathrm{ij}}^{⬚}$ and $\beta_{\mathrm{ij}}^{0}$ are the stoichiometric (or conditional) and thermodynamic equilibrium constant, respectively. Both EDH and SIT models assume that the variation of the activity coefficients (*γ*) with charge (z) and ionic strength (*I*) can be expressed by the general formula:

log *γ* = −z^2^·A · *I*^0.5^/(1+1.5·*I*^0.5^) + *f* (*I*) (4)

(5) $$A=\left(0.51+\frac{0.856\;\cdot \;\left(T\;-\;298.15\right)+0.00385\cdot {\left(T\;-\;298.15\right)}^{2}}{1000}\right)$$

where *f*(*I*) is a term accounting for ionic strength dependence. Rearranging Equation (3) according to Equation (4) leads to:

(6) $${\log\beta }_{\mathrm{ij}}{=\log\beta }_{\mathrm{ij}}^{0}\;-\;0.51\;\cdot {\;z}^{*}\cdot \;D.H.+f\left(I\right)$$

(7) $$D.H.=\frac{\sqrt{I}}{1+1.5\;\cdot \;\sqrt{I}}$$

(8) $$z^{*}=\Sigma \;(\mathrm{charge}{{)}^{2}}_{\mathrm{reactant}}\;-\;\Sigma \;(\mathrm{charge}{{)}^{2}}_{\mathrm{product}}$$

Equation (5) comes from the polynomial fitting of tabulated values of A at different temperatures.

The differences between the two models stem from the nature of the *f*(*I*) term as well as from the adopted concentration scale, namely molar for the EDH equation, Equation (9), and molal the SIT model, Equation (10):

(9) $${\log\beta }_{\mathrm{ij}}{=\log\beta }_{\mathrm{ij}}^{0}\;-\;A\;\cdot {\;z}^{*}\cdot \;D.H{.+C}_{\mathrm{ij}}\;\cdot \;Ic$$

(10) $${\log\beta }_{\mathrm{ij}}{=\log\beta }_{\mathrm{ij}}^{0}\;-\;A\;\cdot {\;z}^{*}\cdot \;D.H.+\Delta \varepsilon \mathrm{ij}\;\cdot \;Im$$

In the SIT model, Δ*ε*_ij_ is the combination of the specific interaction coefficients of the species involved in the equilibrium and the ions of the supporting electrolyte. For example, in the case of Equation (3) in NaCl_(aq)_, it is:

(11) $$\Delta \varepsilon_{\mathrm{ij}}=i\;\cdot \;\varepsilon (H^{+},\;{\mathrm{Cl}}^{-})+j\;\cdot \;\varepsilon (Mn^{+},\;{\mathrm{Cl}}^{-})+\varepsilon ({\mathrm{Na}}^{+},\;L4^{-})\;-\;\varepsilon ({\mathrm{Na}}^{+},M_{j}H_{i}L^{\left(i+\mathrm{jn}-4\right)})$$

When a neutral species, such as the M_2_L species for a divalent metal cation, is involved, Equation (11) becomes:

(12) $$\Delta \varepsilon_{02}=2\;\cdot \;\varepsilon \;(M2^{+},{\mathrm{Cl}}^{-})+\varepsilon \;({\mathrm{Na}}^{+},L4^{-})\;-\;k_{m}(M_{2}L)$$

where *k_m_* is the Setschenow coefficient.

The dependence of the equilibrium constant on temperature was modeled by the van’t Hoff equation, as follows:

(13) $${\log\;\beta }_{\mathrm{ij}}{=\log\;\beta }_{\mathrm{ij}\;\theta }+\frac{\Delta H_{\mathrm{ij}}}{R\cdot \ln\left(10\right)}\;\cdot \left(\frac{1}{\theta }\;-\;\frac{1}{T}\right)$$

where *θ* is the reference temperature (*θ* = 298.15 K in our case) and $\Delta H_{\mathrm{ij}}$ is the enthalpy variation of the equilibrium, whose dependence on ionic strength is given by:

(14) $$\Delta H_{\mathrm{ij}}=\Delta H_{\mathrm{ij}}^{0}{-\;z}^{*}\cdot A^{\prime }\;\cdot \;\frac{\sqrt{I}}{1+1.5\cdot \sqrt{I}}+\Delta \varepsilon_{\mathrm{ij}}^{\prime }\cdot \;I\;$$

where $\Delta H_{\mathrm{ij}}^{0}$ is the enthalpy variation of the equilibrium at infinite dilution, and $\Delta \varepsilon_{\mathrm{ij}}^{\prime }$ is the ionic strength dependence parameters of $\Delta H_{\mathrm{ij}}^{0}$; $A^{\prime }$ and $\Delta \varepsilon_{\mathrm{ij}}^{\prime }$ are related to A and $\Delta \varepsilon_{\mathrm{ij}}$ as follows:

(15) $$A^{\prime }{=RT}^{2}\cdot \ln\;\left(10\right)\cdot \frac{\partial A}{\partial T}=1.5+0.024\;\cdot \;\left(T\;-\;298.15\right)$$

(16) $${\Delta \varepsilon }_{\mathrm{ij}}^{\prime }{=RT}^{2}\cdot \ln\;\left(10\right)\cdot \frac{\partial \Delta \varepsilon }{\partial T}$$

Combining Equation (10) with Equation (14), the equation to which the experimental data were fitted is:

(17) $${\log\;\beta }_{\mathrm{ij}}{=\log\;\beta }_{\mathrm{ij}\;\theta }^{0}{-\;z}^{*}\cdot \;A\;\cdot \frac{\sqrt{I}}{1+1.5\cdot \sqrt{I}}{+\Delta \varepsilon }_{\mathrm{ij}}\;\cdot \;I+\frac{\Delta H_{\mathrm{ij}}^{⬚}}{R\cdot \ln\left(10\right)}\;\cdot \left(\frac{1}{\theta }-\;\frac{1}{T}\right)$$

Conventionally, thermodynamic parameters refer to the molal concentration scale, and the symbolism referred to in the molar concentration scale has the superscript “^M^” (e.g., the protonation enthalpy change at infinite dilution in the molar concentration scale is indicated by $\Delta H_{i0\;\theta }^{0\;M}$).

The sequestering ability of GLDA toward Cd^2+^ is computed by calculating pL_0.5_. This semi-empirical parameter allows comparisons among ligands as it eliminates the contribution of ligand protonation constants, metal cation hydrolysis constants and any other reaction (e.g., chloride complexes) competing with it under analysis, namely the formation constants of the M/L system. Moreover, pL_0.5_ does not depend on the speciation scheme, but only on the experimental conditions (pH, ionic strength, and temperature) used for its computation.

Briefly, the higher the pL_0.5_ is the stronger the sequestering ability. The reader may find further details concerning pL_0.5_ definition and use in [38,39,40,41,42].

## 3. Results and Discussion

For reasons of transparency and to report the real experimental data of this study, the data couples represented by the cm^3^ of titrant vs. e.m.f. obtained for all the potentiometric titrations analyzed in this work are reported as Supplementary Materials (Excel spreadsheet). The pairs of values represent all the recorded points, but not all the recorded points have necessarily been taken into consideration during the data analysis, as different pH values were explored.

### 3.1. Acid–Base Properties of L-glutamic-N,N-diacetic Acid

The acid–base properties of the L-glutamic-N,N-diacetic acid were obtained in the molar concentration scale at different ionic strengths, temperatures and various ionic media. Conditional protonation constant values were then converted to a molal concentration scale as reported in the experimental section. As the amount of data is too large, the full record of the values obtained, both in the molar and in the molal concentration scales, is reported as Supplementary Materials (Table S1).

The data analysis, according to all models mentioned, was performed using the protonation constants reported in this work at different temperatures and ionic strengths in aqueous NaCl, KCl, and (C_2_H_5_)_4_NI solutions and those already published by Bretti et al. [43] at 298.15 K in aqueous NaCl, (CH_3_)_4_NCl and (C_2_H_5_)_4_NI at *I* ≤ 2.134 mol kg^−1^. This last choice was required to fit protonation data as in Equation (9), with the consideration that the term *f*(*I*) is linear, whereas Bretti et al. [43] used a two-parameter approach.

**Figure 1:**
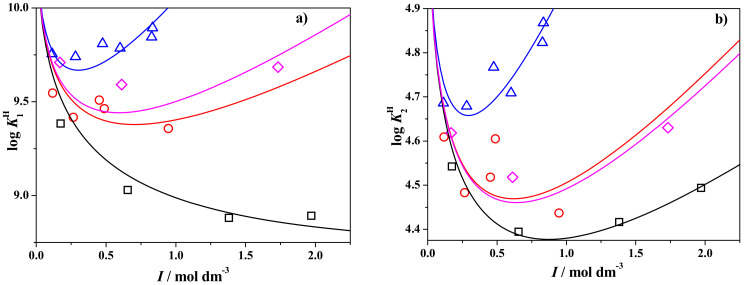
Dependence of the first (**a**) and second (**b**) protonation constants of GLDA on ionic strength at *T* = 298.15 K in NaCl_aq_ (□), KCl_aq_ (○), (CH_3_)_4_NCl_aq_ (◊) and (C_2_H_5_)_4_NI_aq_ (∆).

The trend observed for the protonation constants values, especially regarding the HL and H_2_L species, is reported in Figure 1 and it traces what has already been discussed by Bretti et al. [43] as values in (C_2_H_5_)_4_NI are higher than those in (CH_3_)_4_NCl, which in turn, is higher than KCl and NaCl. This behavior indicates that sodium cation can interact with the fully deprotonated and the monoprotonated GLDA anion more when compared to the potassium cation and tetramethylammonium, whereas tetraethylammonium seems to be the cation less able to interact among those used as the background electrolyte. The continuous line depicted in Figure 1 represents the fitting to the EDH model.

For the H_3_L and H_4_L species, whose values are much lower compared to the first two protonation constants, the trend is less evident.

The trend discussed is well-established regarding log *K*^H^_1_ with relevant differences among ionic media, i.e., the whole stability constant values are spread within one order of magnitude in terms of the protonation constant. Contrarily, differences among log *K*^H^_2_ (Figure 1b) are less important and in some cases, such as in the comparison between KCl and (CH_3_)_4_NCl, are within the experimental errors.

Once the protonation constants, both in the molar and in the molal concentration scales are provided, the data analysis was performed with the EDH, SIT and pure water model approaches. Regarding the EDH and SIT approaches, the whole set of conditional protonation constants (Table S2) was fitted to Equation (17) and a set of parameters useful for calculating the protonation constants of GLDA at any value of temperature and ionic strength within the experimental domain was obtained (Table 3). During this fitting procedure, the values of the protonation constants at infinite dilution and *T* = θ = 298.15 K (log $\beta_{i0\;\theta }^{0}$) were fixed in accordance with those already published by Bretti et al. [43].

The parameters reported in Table 3 show some trends, for example, the values of *C*_i_ within the same ionic medium decrease with increasing the number of protons of the species. The only exception to this trend is represented by the *C*_1_ reported for NaCl_aq_.

Regarding the values of protonation enthalpy changes, the value found for the first step, namely $\Delta H_{1\;\theta }^{0}$ = (−11.6 ± 1.4) kJ mol^−1^, is slightly less negative compared to other values reported in the literature for the interaction of a proton with a ternary amine; for example, $\Delta H_{1\;\theta }^{0}$ = −25 kJ mol^−1^ for methylglycindiacetic acid [44], $\Delta H_{2\;\theta }^{0}$ = −19 kJ mol^−1^ for risedronic acid [45], $\Delta H_{1\;\theta }^{0}$ = −23 kJ mol^−1^ for ethylenediaminetetracetic acid [46] and $\Delta H_{1\;\theta }^{0}$ = −19 kJ mol^−1^ for nitrilotriacetic acid was found [46]. Compared to the parent glutamic acid molecule [47], the protonation constant at infinite dilution of *Glda*^4−^ is slightly higher (log $K_{10\;\theta }^{0}$ = 10.56 and 10.01, for *Glda*^4−^ and glutamate, respectively), whereas the protonation enthalpy is significantly lower ($\Delta H_{10\;\theta }^{0\;M}$ = −12 and −40, same order), resulting in different thermodynamic behavior for proton binding, being enthalpically-driven for glutamate and entropically-driven for *Glda*^4−^.

Using the data listed in Table 3 in the molal concentration scale, thermodynamic parameters (∆*G*^0^, ∆*H*^0^, and *T*∆*S*^0^, in kJ mol^−1^) were calculated at different ionic strength values. The data given in Table 4 reveal that the first (relative to the amino group) and the fourth protonation steps are exothermic in all conditions; the second and the third steps are slightly endothermic at low ionic strength values and tend to become less positive at *I* ≥ 0.25 mol kg^−1^ or negative in the case of sodium chloride. The contribution to the proton binding is generally entropic in nature for the first, second and third protonation steps in all ionic media and at all ionic strength values. Conversely, the fourth protonation step is enthalpic in nature with this trend even more evident at *I* ≥ 0.75 mol kg^−1^ in NaCl_aq_. Similar behaviors were observed in the literature for molecules with the same functional groups (e.g., [38,44]).

Experimental protonation constants (Table S2) were also analyzed by the “pure water model”. This approach is based on a chemical model, in which the different ionic strength dependence of conditional protonation constants observed in different ionic media are dependent on the chemical interaction taking place between the ligand (L) and the ions of the supporting electrolyte (e.g., NaCl), through the formation of several “weak species”, such as NaL. The aim of this approach is to split the conditional protonation constants to obtain “pure” protonation constants valid in any ionic media and weak species relative to the specific cation or anion interacting with the ligand. The ionic strength dependence of both protonation constants and weak species is a function only of the reaction stoichiometry within a reasonably narrow ionic strength range (0 < *I*/mol L^−1^ ≤ 1.0). The chemical speciation of a ligand in a multi-component solution must be drawn considering the “pure” protonation constants and the weak species relative to all the components of the solution calculated at the ionic strength of such solution and considering the concentration of all the interacting ions. Theoretical bases about this approach are available in the literature, including the requirements of using the molar concentration scale, both for ionic strength and equilibrium constants [48,49], the presence of a background electrolyte not—or very weakly—interacting with the ligand (generally tetraethylammonium iodide), and the ionic strength range not exceeding *I* = 1.0 mol dm^−3^ [50,51,52,53]. Some successful examples of applications of this model to other molecules are reported in the literature (e.g., [44,45,54]), where the readers may also find extensive discussion about the fitting ability and the analogy with the hybrid SIT model in terms of the chemical information carried.

As already mentioned, according to the pure water model the ionic strength dependence of the equilibrium constants is just a function of the stoichiometric coefficients and it is described by the following Equation:

(18) $$\log\;K=\log\;K^{0}\;-\;z^{*}\;\cdot \;(I^{0.5}/(2\;+\;3\;I^{0.5}))\;+\;\;\cdot \;(c_{0}\;\cdot \;p^{*}\;+\;c_{1}\;\cdot \;z^{*})\;-\;0.1\;\cdot \;z^{*}\;\cdot \;I^{1.5}$$

(19) $$p^{*}=\;\Sigma \;(\mathrm{stoich}.\;\mathrm{coeff}{)}_{\mathrm{reactants}}\;-\;\Sigma \;(\mathrm{stoich}.\;\mathrm{coeff}{)}_{\mathrm{products}}$$

where log *K* can be the protonation constant (*K*^H^) or the weak complex formation constant (*K*_MjHiL_), log *K*^0^ is the same quantity at infinite dilution, *c*_0_ and *c*_1_ are the ionic strength dependence parameters common to all the species, and z* is given in Equation (8).

Whenever data at different temperatures are available, adjustable parameters of Equation (18) (i.e., log *K*^0^, *c*_0_, *c*_1_) can be written as follows:

(20) $$Y_{T}{=Y}_{\theta }+\frac{\partial Y}{\partial T}\cdot \left(T\;-\;\theta \right)$$

(21) $$\frac{\partial \log\;K}{\partial T}=\frac{\Delta H_{i0\;\theta }^{0\;M}}{{RT}^{2}\ln(10)}\approx \;\frac{\Delta H_{i0\;\theta }^{0\;M}}{1702}\;(\mathrm{at}\;T=298.15\;K)$$

where Y_T_ is the value of the parameters at the temperature *T*, Y_θ_ is the value at the reference temperature θ (298.15 K) and the partial derivative accounts for the temperature dependence. The first derivative parameter originates from the expansion to the Taylor series of the parameter Y approximately at the point θ. For Y = log *K* this is the first derivative on the temperature of Equation (13), which in turn corresponds to $\Delta H_{i0\;\theta }^{0\;M}$ divided by R*T*^2^ ln (10) (Equation (21)).

To avoid misinterpretations, protonation constants at infinite dilution were kept fixed to values reported in Table 3 (in the molar concentration scale) and the value of the partial derivative accounting for the temperature dependence of log *K* was obtained dividing the value of $\Delta H_{i0\;\theta }^{0\;M}$ reported in Table 3 by the value of 1702 (Equation (21)). Moreover, to bypass the correlation between *c*_0_ and *c*_1_ parameters of Equation (18) during the fitting procedure, it was chosen to refine only one of them. Since values of z* are higher than those of p* (see Table 5), it was preferred to refine *c*_1_ and to fix the value of *c*_0_ at *c*_0_ = 0.165.

The conditional protonation data in the molar concentration scale at different ionic strengths and temperatures and in different ionic media were analyzed with ES2WC software [30], the best fit was obtained by refining the equilibrium constants of thirteen weak species, namely NaL, Na_2_L, NaHL, NaH_2_L, NaH_3_L, KL, KHL, KH_2_L, KH_3_L, (CH_3_)_4_NL, (CH_3_)_4_NHL, (CH_3_)_4_NH_2_L and (CH_3_)_4_NH_3_L. A summary of all the parameters refined is given in Table 5.

Data reported in Table 5 indicate that the ligand anion (L^4−^) interaction with the sodium cation is stronger than that with the potassium and tetramethylammonium cations. In fact, the formation constant values of the ML species at infinite dilution are log *K* = 2.50, 2.20 and 1.89, respectively. Moreover, for the sodium cation only, the presence of a dinuclear M_2_L species was evidenced. The stepwise formation constant value of this species can be obtained by subtracting the value of the NaL from that of the Na_2_L, thus, is log *K* = 3.01 − 2.50 = 0.51. Similarly, the stepwise formation constant values of the protonated species can be obtained by subtracting the values of the protonation constants from those of the MH_i_L species. Values so obtained decrease linearly with the increasing number of the protons of the species, the slopes obtained for the three cations are not statistically different from each other. Additionally, in this case, similar trends have been observed in the literature [52,55].

### 3.2. Interaction of L-glutamic-N,N-diacetic Acid with Cd^2+^

Interaction of L-glutamic-N,N-diacetic acid with Cd^2+^ was studied by means of potentiometric titrations at different ionic strength values and at *T* = 298.15 K in aqueous NaCl solutions. The data analysis was performed using the BSTAC4 computer program to analyze titrations carried out at different ionic strength values to obtain the formation constants of the Cd_j_H_i_L species at infinite dilution together with the relative ionic strength dependence parameters of Equation (9), thus, no experimental data at different ionic strength values are available. During this process, the protonation constants of *Glda*^4−^ relative to the NaCl medium were kept fixed to the values reported in Table 3; the equilibrium constants of the Cd^2+^/Cl^−^ and Cd^2+^/OH^−^ species were taken from [56]. The results of the data analysis performed in the pH range 2 ≤ pH ≤ 11 are summarized in Table 6, reporting the formation constant at infinite dilution (log $\beta_{\mathrm{ij}}^{0}$) of the found species, the ionic strength dependence parameters (*C*_ij_), the maximum of formation of each species (Max (%)) and the pH value at which it was achieved.

The choice of the best speciation model is always a challenging task. In this work, a statistical approach based on the variance ratio and goodness-of-fit (GOF) criteria was used for this purpose. According to Crea et al. [57], the criteria used to select the chemical model are: (a) the value of the variance ratio among different speciation models; (b) the simplicity of the model (other minor species can be added to the chosen ones but the model becomes unrealistically complicated); (c) the likelihood of the proposed species, in particular, in relation to similar systems.

The mean deviation of the whole fit (i.e., the average deviation of the experimental e.m.f. of each datum point, in absolute value, from the fitted value) is 3.7 mV. This value is acceptable for a complex system, also considering the variable ionic strength and the wide pH range considered.

The speciation model reported in Table 6 seems to be the most likely for the system under analysis. Together with these species reported, the possible presence of the M_3_L species was also tested (log $\beta_{03}^{0}$ =21.92 ± 0.05 and C_03_ = 2.33 ± 0.11) but the formation percentage reached was significant (~15%) only in a few titrations with a high *C*_Cd_:*C*_GLDA_ ratio to include it in the final speciation scheme. Moreover, limiting the pH to the acidic range (2.0 < pH < 5.0), several minor species were determined. Some examples of possible speciation schemes refined in the mentioned pH range are reported as Supplementary Materials (Table S2) and also contain polynuclear species such as M_2_H_3_L, M_2_H_2_L_2_, M_2_H_3_L_3_, M_3_H_2_L_2_, M_3_H_3_L_3_, M_3_H_4_L_3_, M_3_H_2_L_4_ and M_4_H_2_L_4_. Interestingly, the value of the stability constant of the ML species obtained in such trials is always considerably lower than that refined in the selected speciation scheme because the value was refined in a pH range where the formation of this species never reaches the maximum, thus, is underestimated.

Although their presence in the final speciation scheme was not taken into account, the formation of the M_3_L and other minor polynuclear species mentioned cannot be excluded. However, considering the stoichiometry of the species, their presence is not fundamental for the assessment of speciation in natural fluids because their formation may occur only in experimental conditions hardly found in nature, namely *C*_Cd_ > *C*_GLDA_ and total concentrations exceeding 2.0 mmol dm^−3^. In any case, the identification of polynuclear species is always an interesting task, but dedicated experiments with proper techniques (e.g., ESI-MS) should be performed to confirm or exclude the presence of such species.

**Figure 2:**
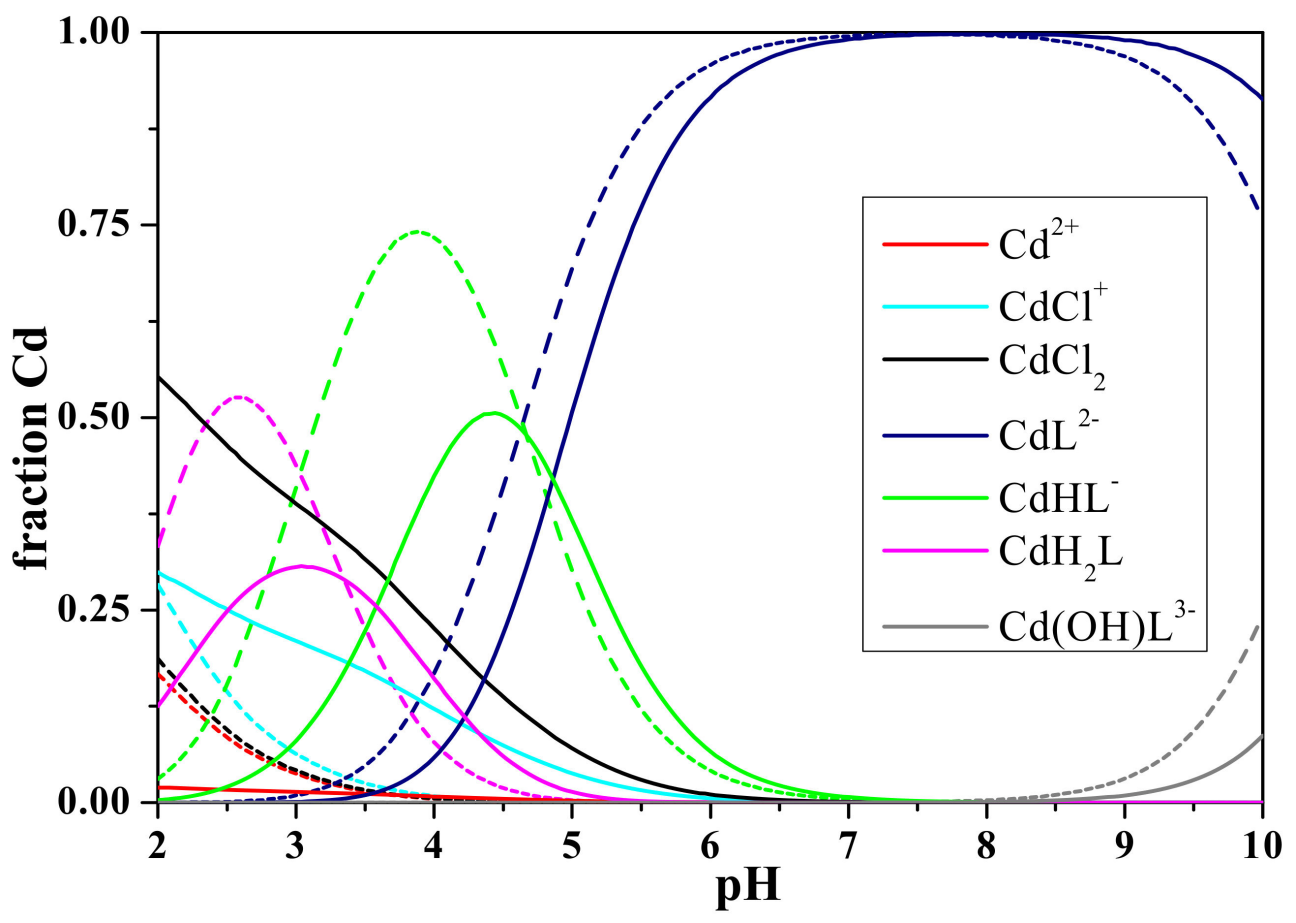
Speciation diagram of the Cd^2+^/*Glda*^4−^ system is given at low ionic strength (*I* = 0.05 mol kg^−1^, dashed line) and at ionic strength of seawater (*I* = 0.72 mol kg^−1^, solid line) at *T* = 298.15 K and *p* = 0.1 MPa. Experimental conditions: *C*_Cd_ = 10^−5^ mol kg^−1^; *C*_L_ = 10^−3^ mol kg^−1^. Species: Cd^2+^ (red), CdCl^+^ (cyan), CdCl_2_^0^_(aq)_ (black), CdL^2−^ (navy), CdHL^−^ (green), CdH_2_L^0^_(aq)_ (magenta), Cd(OH)L^3−^ (gray).

The importance of the complex formation constants of the Cd^2+^/*Glda*^4−^ species is depicted in Figure 2, where the speciation diagram of the system is given at low ionic strength (*I* = 0.05 mol kg^−1^, dashed line) and at the ionic strength of seawater (*I* = 0.72 mol kg^−1^, solid line). At low ionic strength values, the importance of the chloro complexes is marginal so that the complexation with the ligand is more relevant. Moreover, as ionic strength increases the complexation is shifted to more alkaline pH values by ~ 0.5 pH units. In the experimental conditions selected (*C*_Cd_ = 10^−5^ mol kg^−1^; *C*_L_ = 10^−3^ mol kg^−1^), the CdH_2_L^0^_(aq)_ species reach the maximum formation percentages at pH = 2.63 (52 %) and pH = 3.07 (31 %) at *I* = 0.05 and 0.72 mol kg^−1^, respectively, whereas for CdHL^-^ it is at pH = 3.92 (74 %) and pH = 4.43 (50 %) in the same conditions. This also applies to the CdL^2−^ species, but the formation percentage reaches 100 % in both cases, and to the Cd(OH)L^3−^ species, even if the maximum of formation is not reached in the selected pH range. The Cd_2_L^0^_(aq)_ species is not formed in these experimental conditions.

**Figure 3:**
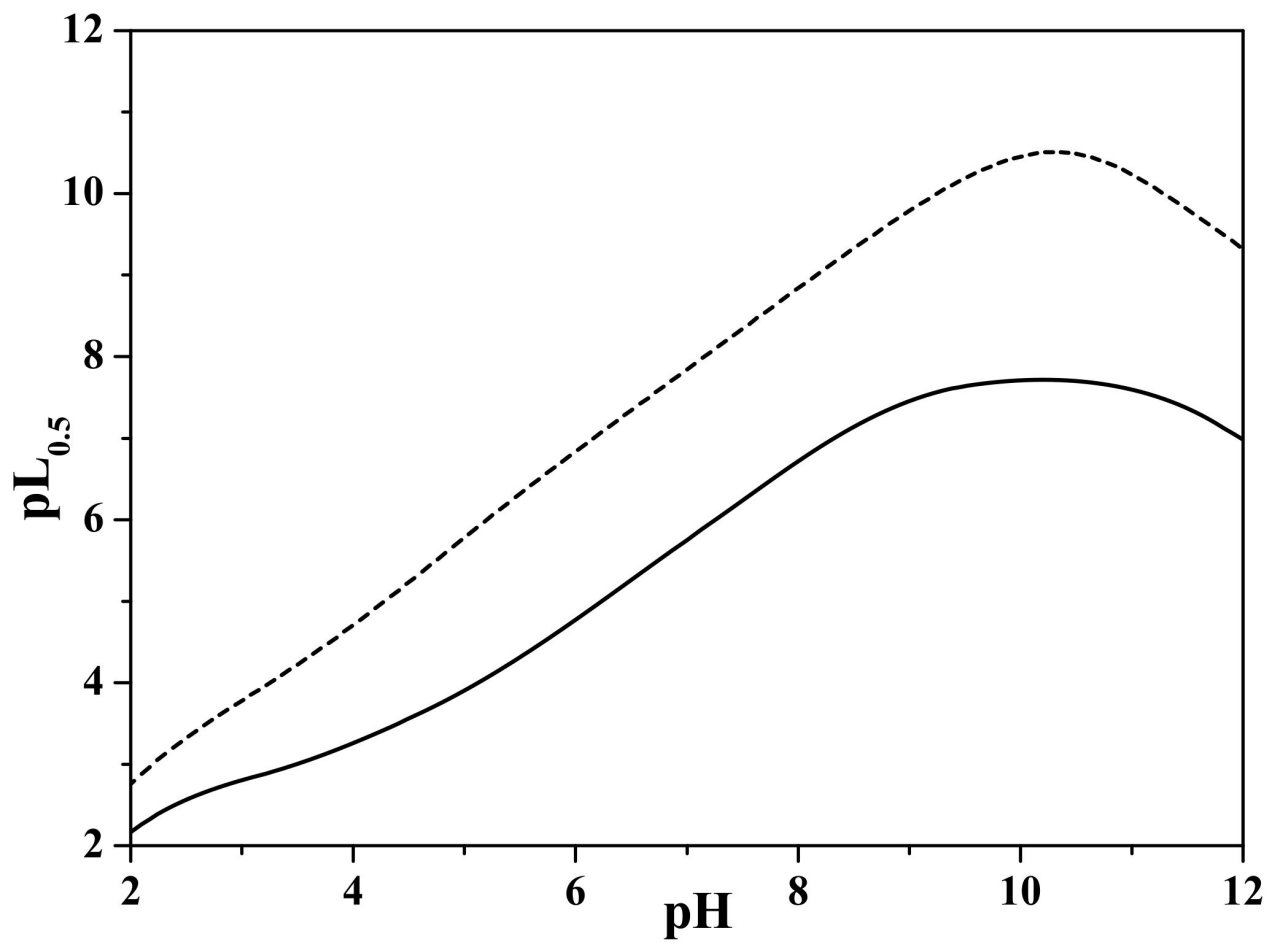
Dependence of the pL_0.5_ in pH at *I* = 0.05 mol kg^−1^ (dotted line) and *I* = 0.72 (solid line) mol kg^−1^ at *T* = 298.15 K and *p* = 0.1 MPa.

Apart from the numerical value of the equilibrium constants, an operational measure to quantify the sequestering ability and, therefore, select the most suitable chelating agent for the sequestration of a given metal cation is required. Many parameters have been proposed and most of them are reviewed by Bazzicalupi et al. [58] and Gama et al. [59]. In this work, the sequestering ability of GLDA toward Cd^2+^ is assessed by computing pL_0.5_. The value of the pL_0.5_ represents the antilogarithm of the total ligand concentration required to sequester 50 % of the metal cation present as the trace level in each condition. Crea et al. [39] pointed out the similarity of pL_0.5_ and the Schwarzenbach’s (*K*ʹ or apparent, *K*_app_) constant; in this light, it is noticeable that the shape of the function reported in Figure 3 is very similar to that reported in the technical report by AkzoNobel, computed starting from NIST data [60], for the apparent formation constants (i.e., Schwarzenbach) of other cations with weak hydrolysis, such as Mg^2+^ and Ca^2+^. However, the use of pL_0.5_ has some relevant advantages over the apparent constants, especially in very complex systems. Among them, it is significant that it is easier also to be determined by non-specialist users by means of dedicated computer programs, without any deep knowledge of the treatment of simultaneous equilibria and mass balance equations.

The dependence of pL_0.5_ of GLDA toward Cd^2+^ on pH at *I* = 0.05 (dotted line) and 0.72 (solid line) mol kg^−1^ is displayed in Figure 3. It can be noted that pL_0.5_ increases almost linearly with pH at both ionic strength values, inverting the trend at pH ~10 due to the hydrolysis of the Cd^2+^ cation. Tracing what was found for the formation percentage of complex species noted in Figure 2, complexation is generally greater at low ionic strength values; in terms of pL_0.5_ it may be noted that values at *I* = 0.05 mol kg^−1^ are higher than those at *I* = 0.72 mol kg^−1^ by one or two orders of magnitude. In this context, it should be noted that experimental data were analyzed up to pH = 10; no information about further complex species with stoichiometry M_j_(OH)_2_L, whose formation occurs at pH > 10, are available, but their presence seems very unlikely in the case of Cd^2+^.

### 3.3. Comparisons with the Literature Data

Minor discrepancies reported for protonation constants have already been discussed, and data are sufficient to assess a robust dependence on temperature and ionic strength.

Regarding the Cd^2+^/*Glda*^4−^ complex formation constants, direct formation constant comparisons with the ones already published can hardly be made since so few values were reported in the literature, and typically only in a single experimental condition. For example, Begun et al. [7] indicated the formation of four complex species, namely CdL^2−^, CdHL^−^, CdH_2_L^0^_aq_ and Cd(OH)L^3−^, in KCl_aq_ at *I* = 0.1 mol dm^−3^ and *T* = 298.15 K, but all the data were obtained by working in only one experimental condition of equimolar concentration of metal cation and GLDA (1 mmol dm^−3^). Although the four cited species were also found in the current work, the formation constant values suffer from some numerical discrepancies likely deriving from the different ionic mediums and, thus, from the different values of the protonation constants used. Moreover, from the distribution diagram reported by Begun et al. [7], it seems that the Cd^2+^/Cl^−^ species were not considered in the speciation model so that the formation constant values for the Cd^2+^/*Glda^4^*^−^ species must be considered as conditional. Besides the mentioned differences in the evaluation of equilibrium constants, distribution diagrams reported in Figure 2 are comparable to the ones found by Begun et al. [7] but only if, in our case, the formation percentages of the Cd^2+^, CdCl^+^ and CdCl_2_^0^_(aq)_ are summed up.

**Figure 4:**
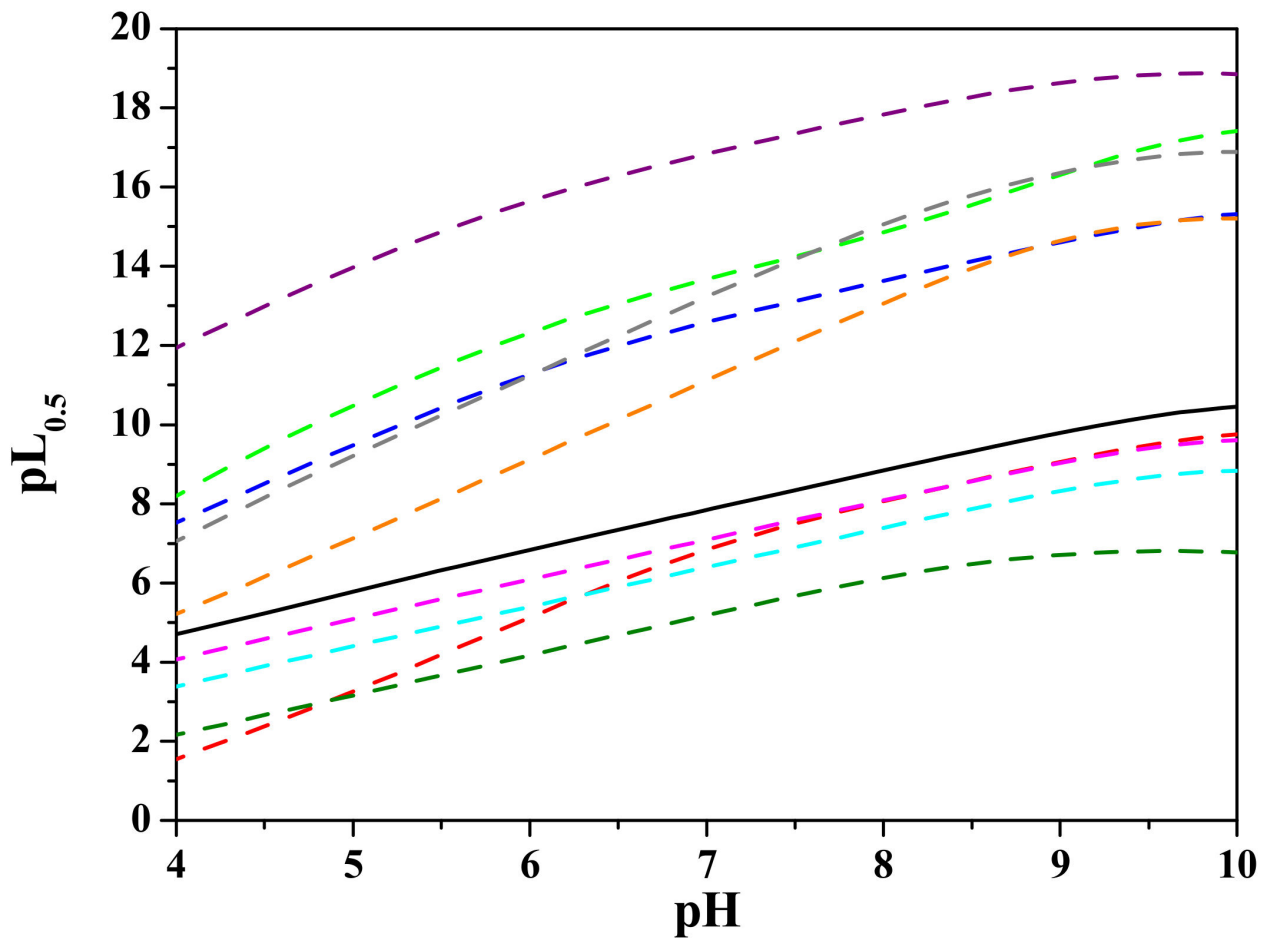
Comparison of the pL_0.5_ of various complexones toward Cd^2+^ at *I* = 0.1 mol dm^−3^ and *T* = 298.15 K at different pH values. Complexones: EDDS (red), TTHA (green), EDTA (blue), DTPA (gray), MGDA (magenta), NTA (cyan), EGTA (orange), CDTA (purple), HIDA (olive), GLDA (black).

By taking from the literature the most reliable data for the interaction of Cd^2+^ with some complexones, such as ethylendiaminetetracetic acid (EDTA [61]), triethylenetetraminehexaacetic acid (TTHA, [62]), ethylenediamine-N,N’-disuccinic acid (S,S-EDDS, [63]), N-(2-hydroxyethyl)iminodiacetic acid (HIDA, [64]), nitrilotriacetic acid (NTA, [65]), Cyclohexanediaminetetraacetic acid (CDTA, [60]), diethylenetriaminepentaacetic acid (DTPA, [66]), ethylene glycol-bis(β-aminoethyl ether)-N,N,N′,N′-tetraacetic acid (EGTA, [67]) and methylglycindiacetic acid (MGDA, [68]) the pL_0.5_ was computed in the same conditions of temperature (*T* = 298.15 K) and ionic strength (*I* = 0.1 mol dm^−3^) in the pH window of natural waters (4 ≤ pH ≤ 10, Figure 4). The plots obtained are almost parallel, depending on the protonation constants of the complexone, with a maximum between pH ~9 and pH ~10. Complexones with more binding sites display higher pL_0.5_ values, as in the case of TTHA (ten binding sites) and DTPA (eight binding sites). Quite surprisingly, CDTA (with six binding sites) shows the highest pL_0.5_ values among all complexones. GLDA, with five binding sites and depicted with a solid line, has an average value between MGDA (four binding sites) and EGTA (six binding sites). Interestingly, among the new generation biodegradable chelants, namely MGDA, NTA, EDDS and HIDA, GLDA achieves the highest pL_0.5_ value.

## 4. Conclusions

Although the industrial employment of GLDA as a sustainable and biodegradable new generation chelating agent seems promising as an alternative to the traditional chelants, the scientific literature on this topic is still not in agreement. With the aim of providing a solid foundation for the development of sustainable strategies for water and soil remediation, here we report a systematic study on GLDA protonation thermodynamics and interaction with Cd^2+^ in an aqueous solution. New data in a standard state (i.e., infinite dilution) are given here, together with parameters useful for calculating protonation thermodynamics, the formation constants and the sequestering ability of GLDA toward Cd^2+^ at the pH and ionic strength values of most natural fluids. From the data discussed here, it can be claimed that a deprotonated ligand is involved in four successive protonation steps, the first being related to the nitrogen atom and the following to the carboxylate groups. In addition, the medium effect was interpreted by means of the so-called “pure water model”; according to this approach, the dependence on ionic strength in different media depends on the formation of weak species between the ligand, or its partially protonated forms, with the cations of the supporting electrolyte. The interaction of the *Glda*^4−^ with Na^+^ results were greater with respect to the ones displayed by K^+^ and (CH_3_)_4_N^+^, and a set of thirteen weak complexes, with temperature and ionic strength coefficients, was obtained.

The whole of the data collected on the Cd^2+^/*Glda*^4−^ system made it possible to determine a speciation scheme featured by five complex species, namely CdL^2−^, CdHL^−^, CdH_2_L^0^_(aq)_, Cd(OH)L^3−^ and Cd_2_L^0^_(aq)_. Furthermore, stability constants at different ionic strength values and at *T* = 298.15 K were assessed. As discussed, the speciation of Cd^2+^/*Glda*^4−^ is largely influenced by ionic strength, as it is the Cd^2+^/Cl^−^ species relevant only in specific conditions (i.e., in acidic solution and at high chloride concentration values). The pL_0.5_ calculations also made it possible to ascertain that the sequestering ability of GLDA toward cadmium cation increases with the pH up to a value of pH ~10 highlighting, on the other hand, that the presence of a background electrolyte, especially chloride, exerts a negative effect on sequestration.

GLDA, among the new generation biodegradable chelants, featuring the highest pL_0.5_ value, is a good alternative to traditional chelants for sustainable Cd^2+^ removal from waters. Therefore, the implementation of GLDA in strategies and/or materials specifically designed for this purpose deserves further investigation.

## Figures and Tables

**Table 1:** Chemicals used in this work, purchased from Sigma-Aldrich. Purity (mass) as stated by the supplier.

Chemical	Formula	CAS n°	Purification	Assay (Mass)
Sodium chloride	NaCl	7647-14-5	NO	≥99%
Potassium chloride	KCl	7447-40-7	NO	≥99%
Tetraethylammonium iodide	(C_2_H_5_)_4_NI	68-05-3	[27]	98%
Hydrochloric acid	HCl	7647-01-0	NO	≥99%
Tetraethylammonium hydroxide	(C_2_H_5_)_4_NOH	77-98-5	NO	~10% ^a^
Sodium hydroxide	NaOH	1310-73-2	NO	≥99%
Potassium phthalate monobasic	C_8_H_5_O_4_K	877-24-7	NO	≥99.95%
Sodium carbonate	Na_2_CO_3_	497-19-8	NO	99.995%
Cadmium chloride hydrate	CdCl_2_ × H_2_O	654054-66-7	NO	≥98%
L-glutamic acid N,N-diacetic acid tetrasodium salt (GLDA)	C_9_H_10_N_1_O_8_Na_4_	51981-21-6	NO	>80% ^b^

^a^ The value refers to the concentration in the solutions. On the dry basis, the assay is ≥0.995 (on the mass basis); ^b^ AkzoNobel declares the product is high purity and NTA free (<0.2 %).

**Table 2:** Summary of the potentiometric titrations performed (L stands for GLDA) performed in different ionic media at different temperatures, ionic strengths, and *p* = 0.1 MPa.

System	Medium	*T*/K	*I* ^a^	*C*_H_^b^	*C*_L_^b^	*C*_M_^b^	n.tit
H^+^/L	NaCl	288.15 and 310.15	0.1 to 1.0	10 to 20	2.5 to 4		24
H^+^/L	KCl	288.15 to 310.15	0.1 to 1.0	10 to 18	2.5 to 4		33
H^+^/L	(C_2_H_5_)_4_NI	288.15 to 310.15	0.1 to 1.0	10 to 20	3.0 to 5		29
Cd^2+^/L	NaCl	298.15	0.1 to 1.0	8.0 to 10	1.0 to 4.0	0.4 to 2.0	30

^a^ mol dm^−3^; ^b^ mmol dm^−3^; standard uncertainties (*u*): *u*(*T*) = 0.1 K; *u*(*I*) = 0.001 mol dm^−3^; *u*(*p*) = 1 kPa; *u*(*C*_H_) = *u*(*C*_L_) = *u*(*C*_M_) = 10^−2^ mmol dm^−3^.

**Table 3:** Parameters obtained fitting experimental protonation constants to Equation (17) in the molar concentration scales at *T* = 298.15 K and *p* = 0.1 MPa.

**Molar Concentration Scale**										
				**NaCl**		**KCl**		**(CH_3_)_4_NCl**	**(C_2_H_5_)_4_NI**	
i	$\log\;K_{i0\;\theta }^{0}$	$\Delta H_{i0\;\theta }^{0\;M}$	z* ^a^	*C*_i0_	$\Delta \varepsilon_{i0}^{\prime \;M}$	*C*_i0_	$\Delta \varepsilon_{i0}^{\prime \;M}$	*C*_i0_	*C*_i0_	$\Delta \varepsilon_{i0}^{\prime \;M}$
1	10.560	−11.6 ± 1.4 ^b^	8	−0.03 ± 0.01 ^b^	8 ± 5 ^b^	0.46 ± 0.02 ^b^	0 ± 3 ^b^	0.55 ± 0.03 ^b^	1.07 ± 0.02 ^b^	−4 ± 3 ^b^
2	5.324	2.0 ± 0.9	6	0.28 ± 0.01	−20 ± 3	0.34 ± 0.02	2 ± 1	0.37 ± 0.02	0.82 ± 0.01	0 ± 2
3	3.071	3.0 ± 1.9	4	0.16 ± 0.02	−13 ± 2	0.16 ± 0.02	1 ± 1	0.27 ± 0.02	0.63 ± 0.02	0 ± 1
4	1.897	−7.0 ± 3.5	2	0.09 ± 0.02	−6 ± 1	0.02 ± 0.09	0 ± 1	0.25 ± 0.01	0.55 ± 0.07	0 ± 1
**Molal Concentration Scale**										
				**NaCl**		**KCl**		**(CH_3_)_4_NCl**	**(C_2_H_5_)_4_NI**	
i	$\log\;K_{i0\;\theta }^{0}$	$\Delta H_{i0\;\theta }^{0}$	z* ^a^	∆*ε*_i0_	$\Delta \varepsilon_{i0}^{\prime }$	∆*ε*_i0_	$\Delta \varepsilon_{i0}^{\prime }$	∆*ε*_i0_	∆*ε*_i0_	$\Delta \varepsilon_{i0}^{\prime }$
1	10.559	−12.0 ± 1.4 ^b^	8	−0.03 ± 0.01 ^b^	8 ± 5 ^b^	0.45 ± 0.02 ^b^	0 ± 3 ^b^	0.44 ± 0.03 ^b^	0.91 ± 0.02 ^b^	−4 ± 3 ^b^
2	5.323	1.7 ± 0.9	6	0.27 ± 0.01	−20 ± 3	0.33 ± 0.02	2 ± 1	0.29 ± 0.02	0.69 ± 0.01	−1 ± 2
3	3.070	3.0 ± 1.9	4	0.15 ± 0.02	−13 ± 2	0.15 ± 0.02	1 ± 1	0.19 ± 0.02	0.49 ± 0.02	−1 ± 1
4	1.896	−6.9 ± 3.5	2	0.08 ± 0.02	−7 ± 1	0.01 ± 0.09	1 ± 1	0.16 ± 0.01	0.44 ± 0.07	0 ± 1

^a^ z* is a parameter of Equation (17); ^b^ ± s (standard deviation); standard uncertainties (*u*): *u*(*T*) = 0.1 K; *u*(*p*) = 1 kPa; *u*(log $\beta_{i0\;\theta }^{0}$) = 0.005.

**Table 4:** Thermodynamic parameters (∆*G*^0^, ∆*H*^0^ and *T*∆*S*^0^, in kJ mol^−1^) for the four GLDA protonation steps (i) at different ionic strength values and *T* = 298.15 K and *p* = 0.1 MPa.

			NaCl			KCl			(C_2_H_5_)_4_NI		
*T*	*I* ^a^	i	−∆*G*^0^	∆*H*^0^	T∆*S*^0^	−∆*G*^0^	∆*H*^0^	T∆*S*^0^	−∆*G*^0^	∆*H*^0^	T∆*S*^0^
298.15	0.00	1	60.3	−12	48	60.3	−12	48	60.3	−12	48
298.15	0.00	2	30.4	2	32	30.4	2	32	30.4	2	32
298.15	0.00	3	17.5	3	21	17.5	3	21	17.5	3	21
298.15	0.00	4	10.8	−7	4	10.8	−7	4	10.8	−7	4
298.15	0.10	1	55.3	−14	41	55.5	−15	41	55.8	−15	41
298.15	0.10	2	26.8	−2	25	26.8	0	27	27.0	0	27
298.15	0.10	3	15.1	0	16	15.1	2	17	15.3	2	17
298.15	0.10	4	9.6	−8	2	9.6	−7	2	9.8	−7	2
298.15	0.25	1	53.6	−13	40	54.3	−15	39	54.9	−16	38
298.15	0.25	2	25.8	−6	20	25.9	0	25	26.4	−1	25
298.15	0.25	3	14.4	−2	12	14.4	2	16	14.9	1	16
298.15	0.25	4	9.3	−9	0	9.2	−7	2	9.8	−8	2
298.15	0.50	1	52.2	−12	40	53.6	−16	37	54.9	−18	37
298.15	0.50	2	25.2	−11	14	25.3	0	25	26.4	−2	24
298.15	0.50	3	14.0	−6	8	14.0	1	15	14.9	0	15
298.15	0.50	4	9.1	−11	−2	8.9	−7	2	10.1	−8	2
298.15	0.75	1	51.4	−11	41	53.4	−17	37	55.4	−20	36
298.15	0.75	2	25.0	−17	8	25.2	0	25	26.8	−2	24
298.15	0.75	3	13.8	−9	5	13.8	1	15	15.2	0	15
298.15	0.75	4	9.0	−13	−4	8.7	−7	2	10.5	−8	3
298.15	1.00	1	50.8	−9	42	53.5	−17	37	56.1	−21	35
298.15	1.00	2	24.9	−22	3	25.3	0	25	27.3	−3	24
298.15	1.00	3	13.7	−12	1	13.7	2	15	15.7	0	15
298.15	1.00	4	9.0	−15	−6	8.6	−7	2	11.0	−8	3

^a^ in mol kg^−1^; standard uncertainties (*u*): *u*(*T*) = 0.1 K; *u*(*I*) = 0.01 mol kg^−1^; *u*(*p*) = 1 kPa; *u*(∆*G*^0^) = 0.2 kJ mol^−1^; *u*(∆*H*^0^) = 1 kJ mol^−1^; *u*(*T*∆*S*^0^) = 1 kJ mol^−1^.

**Table 5:** Equilibrium constants at infinite dilution, temperature and ionic strength dependence parameters of the weak species for GLDA (L) at *T* = 298.15 K and *p* = 0.1 MPa.

Equilibrium	p* ^a^	z* ^a^	$Y_{\theta }\;(\log\;\beta^{0})$	$\frac{\partial Y}{\partial T}$
H^+^ + L^4−^ = HL^3−^	1	8	10.56 ^b^	−0.0068 ^b^
2 H^+^ + L^4−^ = H_2_L^2−^	2	14	15.88 ^b^	−0.0056 ^b^
3 H^+^ + L^4−^ = H_3_L^−^	3	18	18.96 ^b^	−0.0039 ^b^
4 H^+^ + L^4−^ = H_4_L^0^_(aq)_	4	20	20.86 ^b^	−0.008 ^b^
Na^+^ + L^4−^ = NaL^3−^	1	8	2.50 ± 0.02 ^c^	0.017 ± 0.004 ^c^
Na^+^ + H^+^ + L^4−^ = NaHL^2−^	2	14	12.25 ± 0.03	0.013 ± 0.004
Na^+^ + 2 H^+^ + L^4−^ = NaH_2_L^−^	3	18	16.83 ± 0.03	0.007 ± 0.003
Na^+^ + 3 H^+^ + L^4−^ = NaH_3_L^0^_(aq)_	4	20	19.08 ± 0.07	0.003 ± 0.007
2 Na^+^ + L^4−^ = Na_2_L^2−^	2	14	3.01 ± 0.10	0.018 ± 0.012
K^+^ + L^4−^ = KL^3−^	1	8	2.20 ± 0.05	0.004 ± 0.005
K^+^ + H^+^ + L^4−^ = KHL^2−^	2	14	12.12 ± 0.04	−0.004 ± 0.004
K^+^ + 2 H^+^ + L^4−^ = KH_2_L^−^	3	18	16.81 ± 0.03	−0.004 ± 0.004
K^+^ + 3 H^+^ + L^4−^ = KH_3_L^0^_(aq)_	4	20	19.11 ± 0.06	−0.003 ± 0.007
(CH_3_)_4_N^+^ + L^4−^ = (CH_3_)_4_NL^3−^	1	8	1.89 ± 0.08	-
(CH_3_)_4_N^+^ + H^+^ + L^4−^ = (CH_3_)_4_NHL^2−^	2	14	11.82 ± 0.07	-
(CH_3_)_4_N^+^ + 2 H^+^ + L^4−^ = (CH_3_)_4_NH_2_L^−^	3	18	16.45 ± 0.07	-
(CH_3_)_4_N^+^ + 3 H^+^ + L^4−^ = (CH_3_)_4_NH_3_L^0^_(aq)_	4	20	18.59 ± 0.24	-
Parameter			$Y_{\theta }$	$\frac{\partial Y}{\partial T}$
*c*_0_			0.165 ^d^	
*c*_1_			0.200 ± 0.008 ^c^	−0.0011 ± 0.0003 ^c^

^a^p* and z* are parameters of Equation (18); ^b^ Taken from Table 3 and kept constant during calculations; ^c^ s (standard deviation); ^d^ kept fixed during calculations; standard uncertainties: *u*(*T*) = 0.1 K, *u*(*p*) = 1 kPa.

**Table 6:** Overall metal ligand complex formation constants, ionic strength dependence parameters (both in the molar, *C*_ij_, and in the molal concentration scale, ∆*ε*_ij_) and details on the formation percentages (*C*_Cd_ = 0.4 mmol dm^−3^; *C*_L_ = 0.97 mmol dm^−3^, *I* = 0.5 mol dm^−3^) of Cd^2+^/GLDA^4 −^ species for the selected speciation model obtained in the pH range 2.0 < pH < 10.0 at *T* = 298.15 K and *p* = 0.1 MPa.

Equilibrium	z* ^a^	$\log\;\beta_{\mathrm{ij}}^{0}$	*C*_ij_	∆*ε*_ij_	Max (%)	pH
Cd^2+^ + L^4^^−^ = CdL^2^^−^	16	12.68 ± 0.03 ^b^	−0.32 ± 0.06 ^b^	−0.31 ^c^	98	8.6
Cd^2+^ + H^+^ + L^4−^ = CdHL^−^	20	17.61 ± 0.03	0.66 ± 0.06	0.65	50	4.2
Cd^2+^ + 2 H^+^ + L^4−^ = CdH_2_L^0^_(aq)_	22	20.76 ± 0.05	1.81 ± 0.10	1.77	27	2.9
2 Cd^2+^ +L^4^^−^ = Cd_2_L^0^_(aq)_	24	17.52 ± 0.05	1.49 ± 0.07	1.46	5	4.7
Cd^2+^ + H_2_O + L^4−^ = Cd(OH)L^3−^ + H^+^	10	1.77 ± 0.04	−2.10 ± 0.14	−2.05	18	10.0

^a^p* and z* are parameters of Equation (18); ^b^ s (standard deviation); ^c^ error to be associated is the same as the corresponding *C*_ij_ value; standard uncertainties: *u*(*T*) = 0.1 K, *u*(*p*) = 1 kPa, *u*(Max) = 1 %; *u*(pH) = 0.1.

## Data Availability

All the experimental data are reported in the main text or in supporting files. Any other information about data handling may be obtained upon contacting Gabriele Lando glando@unime.it.

## Supplementary Materials

The following are available online. Table S1. Experimental values of the protonation constants of L-glutamic-N,N-diacetic acid (L-GLDA) in different ionic media, at different temperatures and at different ionic strengths. Table S2. Overall metal ligand complex formation constants, ionic strength dependence parameters and fit statistics of Cd^2+^/*Glda*^4−^ system for some proposed speciation models obtained in the pH range 2.0 < pH < 5.0; excel spreadsheet: Data couples (cm^3^ of titrant vs. e.m.f.) obtained for all the potentiometric titrations analyzed in this work.
