# Supplementary material for: Thermodynamic Solution Properties of a Biodegradable Chelant (L-glutamic-N,N-diacetic Acid, L-GLDA) and Its Sequestering Ability toward Cd2+
Source: Molecules. 2021 Nov 23;26(23):7087. doi: 10.3390/molecules26237087 (PMC8659045; doi:10.3390/molecules26237087)
Supplement: Supplementary file 1 [file molecules-26-07087-s001.zip › molecules-1457922 SF.pdf]

molecules

Supplementary File

Thermodynamic Solution Properties of a Biodegradable Chelant (L-glutamic-N,N-diacetic Acid, L-GLDA) and Its Sequestering Ability toward Cd^{2+}

Clemente Bretti [1], Roberto Di Pietro [1], Paola Cardiano [1], Olivia Gomez-Laserna [2], Anna Irto [1], Gabriele Lando [1,*] and Concetta De Stefano [1]

[1] Department of Chemical, Biological, Pharmaceutical and Environmental Sciences, University of Messina, Viale Ferdinando Stagno d'Alcontres 31, I-98166 Messina (Vill. S. Agata), Italy; cbretti@unime.it (C.B.); robdipietro@unime.it (R.D.P.); pcardiano@unime.it (P.C.); airto@unime.it (A.I.); cdestefano@unime.it (C.D.S.)

[2] Department of Analytical Chemistry, University of the Basque Country (EHU/UPV), Barrio Sarriena s/n, E-48080 Leioa, Bilbao, Spain; olivia.gomez@ehu.eus

* Correspondence: glando@unime.it

Table S1. Experimental values of the protonation constants (± 95 % confidence interval) of L-glutamic N,N-diacetic acid (L-GLDA) in different ionic media, at different temperatures and at different ionic strengths.

Medium	T [a]	I_c [b]	I_m [c]	log $K_1^{H}{}_c$	log $K_1^{H}{}_m$	log $K_2^{H}{}_c$	log $K_2^{H}{}_m$	log $K_3^{H}{}_c$	log $K_3^{H}{}_m$	log $K_4^{H}{}_c$	log $K_4^{H}{}_m$
NaCl	288.15	0.116	0.116	9.58 ± 0.01	9.576	4.62 ± 0.01	4.617	2.55 ± 0.01	2.544	1.70 ± 0.04	1.70
	288.15	0.268	0.269	9.35 ± 0.02	9.348	4.55 ± 0.02	4.548	2.51 ± 0.04	2.508	1.61 ± 0.04	1.61
	288.15	0.498	0.503	9.03 ± 0.01	9.03	4.51 ± 0.01	4.506	2.60 ± 0.02	2.592	1.13 ± 0.06	1.13
	288.15	0.981	0.999	8.87 ± 0.01	8.857	4.41 ± 0.01	4.40	2.36 ± 0.02	2.349	1.37 ± 0.02	1.36
	310.15	0.140	0.141	9.34 ± 0.04	9.34	4.56 ± 0.03	4.552	2.53 ± 0.03	2.526	1.27 ± 0.14	1.27
	310.15	0.262	0.265	9.19 ± 0.03	9.182	4.41 ± 0.03	4.401	2.20 ± 0.03	2.192	1.71 ± 0.10	1.70
	310.15	0.503	0.511	8.94 ± 0.03	8.934	4.34 ± 0.03	4.329	2.19 ± 0.03	2.186	1.23 ± 0.20	1.22
	310.15	0.962	0.987	8.68 ± 0.03	8.672	4.27 ± 0.03	4.254	2.01 ± 0.04	2.001	1.10 ± 0.22	1.09
KCl	288.15	0.126	0.127	9.65 ± 0.04	9.648	4.58 ± 0.04	4.578	2.52 ± 0.04	2.518	1.38±0.20	1.38
	288.15	0.247	0.249	9.51 ± 0.02	9.507	4.59 ± 0.02	4.587	2.26 ± 0.08	2.257	1.40±0.39	1.40
	288.15	0.478	0.485	9.51 ± 0.01	9.504	4.46 ± 0.12	4.454	2.37 ± 0.02	2.364	1.45±0.04	1.44
	288.15	0.990	1.019	9.53 ± 0.06	9.518	4.46 ± 0.08	4.448	2.38 ± 0.08	2.368	1.96±0.08	1.95
	298.15	0.118	0.119	9.55 ± 0.01	9.543	4.61 ± 0.01	4.606	2.65 ± 0.01	2.643	1.70±0.20	1.69
	298.15	0.266	0.269	9.42 ± 0.01	9.413	4.48 ± 0.01	4.479	2.50 ± 0.02	2.499	1.81±0.08	1.81
	298.15	0.452	0.459	9.51 ± 0.01	9.502	4.52 ± 0.01	4.511	2.40 ± 0.02	2.396	2.53±0.02	2.52
	298.15	0.488	0.496	9.46 ± 0.02	9.456	4.61 ± 0.02	4.598	2.69 ± 0.04	2.685	2.40±0.04	2.39
	298.15	0.947	0.976	9.36 ± 0.01	9.344	4.44 ± 0.01	4.424	2.54 ± 0.02	2.531	1.32±0.29	1.31
	310.15	0.110	0.111	9.33 ± 0.03	9.326	4.60 ± 0.03	4.596	2.73 ± 0.03	2.726	1.82±0.04	1.82
	310.15	0.110	0.111	9.45 ± 0.03	9.445	4.62 ± 0.03	4.619	2.68 ± 0.03	2.674	1.72±0.04	1.72
	310.15	0.120	0.121	9.44 ± 0.03	9.439	4.56 ± 0.03	4.557	2.53 ± 0.03	2.521	1.76±0.04	1.76
	310.15	0.250	0.253	9.44 ± 0.03	9.429	4.53 ± 0.03	4.526	2.59 ± 0.03	2.58	1.83±0.04	1.82
	310.15	0.490	0.500	9.26 ± 0.03	9.254	4.37 ± 0.03	4.361	2.31 ± 0.04	2.297	0.36±0.39	0.35
	310.15	0.980	1.015	9.24 ± 0.03	9.226	4.39 ± 0.03	4.377	2.41 ± 0.04	2.397	1.06±0.08	1.04
$(C_2H_5)_4NI$	288.15	0.144	0.148	9.80 ± 0.01	9.789	4.72 ± 0.01	4.705	2.65 ± 0.01	2.64	1.70 ± 0.02	1.69
	288.15	0.281	0.297	9.86 ± 0.01	9.84	4.71 ± 0.01	4.684	2.68 ± 0.02	2.657	1.74 ± 0.02	1.72
	288.15	0.525	0.581	9.83 ± 0.01	9.782	4.71 ± 0.01	4.664	2.67 ± 0.01	2.624	1.87 ± 0.02	1.82
	288.15	0.748	0.867	9.87 ± 0.01	9.807	4.76 ± 0.01	4.695	2.73 ± 0.02	2.669	1.87 ± 0.02	1.81
	310.15	0.118	0.121	9.66 ± 0.03	9.644	4.72 ± 0.03	4.708	2.73 ± 0.03	2.714	1.67 ± 0.04	1.66
	310.15	0.290	0.309	9.62 ± 0.03	9.592	4.70 ± 0.03	4.669	2.73 ± 0.03	2.698	1.68 ± 0.04	1.65
	310.15	0.657	0.754	9.60 ± 0.03	9.538	4.68 ± 0.03	4.623	2.69 ± 0.03	2.63	1.66 ± 0.10	1.60
	310.15	0.726	0.845	9.62 ± 0.03	9.556	4.74 ± 0.03	4.677	2.72 ± 0.03	2.649	1.62 ± 0.04	1.55
	310.15	0.739	0.862	9.61 ± 0.03	9.547	4.71 ± 0.03	4.647	2.69 ± 0.03	2.625	1.33 ± 0.04	1.26

A in K;

Table S2. Overall metal ligand complex formation constants, ionic strength dependence parameters and fit statistics of Cd^{2+}/ *Glda*$^{4-}$ system for some proposed speciation models obtained in the pH range 2.0 < pH < 5.0.

Trial	Species	log β^{0}_{ij}	C_{ji}	Max (%)	pH	σ_{fit}	MD_{fit}
1	**ML**	11.09 ± 0.02	0.13 ± 0.04	27.0	5.0	3.9	1.4
	**M_2H_3L**	27.04 ± 0.03	3.14 ± 0.07	39.0	2.3		
	**$M_3H_3L_2$**	45.15 ± 0.02	3.33 ± 0.10	2.7	4.3		
2	**ML**	11.19 ± 0.02	0.60 ± 0.03	35.0	5.0	3.7	1.19
	**MHL**	16.79 ± 0.07	0.26 ± 0.04	20.5	5.0		
	**M_2H_2L**	24.10 ± 0.07	−0.67 ± 0.44	0.1	2.3		
3	**ML**	10.62 ± 0.01	1.37 ± 0.02	31.2	5.0	3.8	1.35
	**$M_2H_2L_2$**	35.14 ± 0.01	2.67 ± 0.03	5.5	5.0		
4	**ML**	10.62 ± 0.01	1.26 ± 0.02	30.0	5.0	3.9	1.40
	**$M_2H_2L_2$ Cl_2**	35.99 ± 0.02	−0.89 ± 0.05	8.0	4.5		
5	**ML**	10.99 ± 0.02	0.02 ± 0.04	25.0	5.0	3.7	1.26
	**M_2HL**	21.06 ± 0.02	1.10 ± 0.06	6.5	5.0		
	**M_2H_3L**	26.04 ± 0.02	4.18 ± 0.06	22.4	2.3		
6	**ML**	10.57 ± 0.01	1.42 ± 0.02	32.0	5.0	4.0	1.50
	**$M_3H_3L_3$**	53.91 ± 0.02	3.90 ± 0.05	1.5	5.0		
7	**ML**	10.96 ± 0.01	0.89 ± 0.03	34.0	5.0	3.6	1.17

	**$M_2H_2L_2$**	35.52 ± 0.02	2.06 ± 0.04	5.9	5.5		
	**$M_2H_3L_2$**	40.04 ± 0.03	−0.52 ± 0.21	0.1	4.0		
**8**	**ML**	10.27 ± 0.02	1.61 ± 0.02	22.0	5.0	4.35	1.65
	**MH_2L_2**	29.22 ± 0.01	0.22 ± 0.04	12.2	5.0		
**9**	**ML**	10.36 ± 0.01	1.57 ± 0.02	27.0	5.0	4.4	1.66
	**$M_2H_3L_3$**	47.50 ± 0.02	1.08 ± 0.04	37.4	4.5		
**10**	**ML**	10.84 ± 0.01	1.06 ± 0.02	34.0	5.0	3.7	1.25
	**$M_3H_4L_3$**	59.14 ± 0.04	2.00 ± 0.21	0.1	4.7		
	**$M_3H_3L_3$**	54.17 ± 0.03	3.54 ± 0.06	5.0	4.7		
**11**	**ML**	10.11±0.03	1.28 ± 0.06	18.0	5.0	5.0	1.90
	**MH_3L**	23.20±0.04	2.01 ± 0.06	24.6	2.3		
**12**	**$M_3H_4L_3$**	59.03 ± 0.03	1.15 ± 0.06	20.4	5.7	5.8	2.30
**13**	**$M_3H_2L_2$**	39.80 ± 0.03	4.16 ± 0.06	24.8	5.3	4.8	1.60
	**$M_3H_3L_4$**	59.64 ± 0.06	0.53 ± 0.06	1.5	5.0		
**14**	**$M_4H_2L_4$**	59.85 ± 0.03	3.95 ± 0.06	17.9	5.3	6.0	2.50
**15**	**$M_3H_2L_4$**	51.63 ± 0.04	0.69 ± 0.08	21.7	5.2	4.8	1.70
	**$M_5H_3L_4$**	73.10 ± 0.02	7.57 ± 0.06	1.7	5.2		
**16**	**ML**	10.89 ± 0.01	1.01 ± 0.02	30.9	5.0	3.7	1.10
	**MHL**	16.44 ± 0.01	0.84 ± 0.04	19.0	4.7		
**17**	**ML**	11.46 ± 0.03	0.25 ± 0.04	35.2	5.0	3.6	1.10
	**MHL**	16.94 ± 0.02	0.15 ± 0.03	22.9	4.6		
	**MH_2L**	20.40 ± 0.03	−0.35 ± 0.03	1.5	3.6		
**18**	**ML**	11.10 ± 0.02	0.85 ± 0.04	39.1	5.0	4.2	1.70
	**M_2HL**	20.61 ± 0.03	2.56 ± 0.08	8.3	4.5		
**19**	**$M_3H_2L_4$**	52.02 ± 0.04	−0.58 ± 0.07	20.2	5.0	6.4	2.70
